# Gate Tunable Relativistic Mass and Berry's phase in Topological Insulator Nanoribbon Field Effect Devices

**DOI:** 10.1038/srep08452

**Published:** 2015-02-13

**Authors:** Luis A. Jauregui, Michael T. Pettes, Leonid P. Rokhinson, Li Shi, Yong P. Chen

**Affiliations:** 1 Birck Nanotechnology Center, Purdue University, West Lafayette, IN 47907; 2 School of Electrical and Computer Engineering, Purdue University, West Lafayette, IN 47907; 3 Department of Mechanical Engineering, University of Texas at Austin, Austin, TX 78712; 4 Department of Physics and Astronomy, Purdue University, West Lafayette, IN 47907; 5 Materials Science and Engineering Program, University of Texas at Austin, Austin, TX 78712

## Abstract

Transport due to spin-helical massless Dirac fermion surface state is of paramount importance to realize various new physical phenomena in topological insulators, ranging from quantum anomalous Hall effect to Majorana fermions. However, one of the most important hallmarks of topological surface states, the Dirac linear band dispersion, has been difficult to reveal directly in transport measurements. Here we report experiments on Bi_2_Te_3_ nanoribbon ambipolar field effect devices on high-κ SrTiO_3_ substrates, where we achieve a gate-tuned bulk metal-insulator transition and the topological transport regime with substantial surface state conduction. In this regime, we report two unambiguous transport evidences for gate-tunable Dirac fermions through π Berry's phase in Shubnikov-de Haas oscillations and effective mass proportional to the Fermi momentum, indicating linear energy-momentum dispersion. We also measure a gate-tunable weak anti-localization (WAL) with 2 coherent conduction channels (indicating 2 decoupled surfaces) near the charge neutrality point, and a transition to weak localization (indicating a collapse of the Berry's phase) when the Fermi energy approaches the bulk conduction band. The gate-tunable Dirac fermion topological surface states pave the way towards a variety of topological electronic devices.

The extraordinary electronic properties of topological insulators^1^,^2^,^3^ (TIs) make them a unique class of materials relevant for applications such as low power electronic devices, spintronics^1^ and fault-tolerant quantum computation^4^,^5^. TIs feature topologically non-trivial surface states, where carriers are massless relativistic particles with linear energy-momentum band dispersion and with spins locked perpendicular to their momentum^2^,^3^. Existence of such helical Dirac fermion surface states have been experimentally confirmed by angle-resolved photoemission spectroscopy (ARPES)^6^,^7^ and scanning-tunneling microscopy^8^. Transport measurements in bulk crystals of TI (such as Bi_2_Te_3_)^9^ have met more challenges to probe the surface states, because of the non-insulating bulk conduction. In order to reduce the bulk contribution and enhance the surface contribution in TI transport, a number of efforts have been made by growing bulk single-crystals with fine-tuned composition^10^,^11^,^12^,^13^, adding compensating dopants^14^,^15^, growing ultrathin films and nanostructures such as nanowires^16^,^17^ or nanoribbons (NRs)^11^,^18^, and using electrical gating^19^,^20^,^21^ to tune the Fermi energy (E_F_). Nanostructures, with a high surface-to-volume ratio, have been shown to enhance the surface conduction contribution. However, in previous reports, unambiguous measurements of the topological surface states (e.g π-Berry's phase and Dirac fermion dispersion) remain challenging. In this work, we combine nanostructures with ultrahigh-κ gate dielectric by placing Bi_2_Te_3_ NRs on SrTiO_3_ (STO) substrates^22^,^23^. We realize the topological transport regime where bulk is insulating and surface substantially contributes to the conduction, and report clear evidence of the Dirac fermion nature of the gate-tunable topological surface states, directly revealing the characteristic linear energy-momentum dispersion via density-dependent effective mass measurements. The excellent gate tunability of E_F_ (from surface states to bulk conduction band) has also enabled us to observe a predicted transition from the usual weak anti-localization (WAL) behavior to weak localization (WL), reflecting a change in the pseudospin texture and Berry's phase of the quantum coherent charge carriers^24^,^25^.

The Bi_2_Te_3_ NRs were synthesized by catalyst-free vapor solid method, following an approach similar to that of Kong et al.^26^ for thin films. NRs are grown out of the plane of the substrate and they are individually transferred to other substrates or support for further studies, using an electrochemically sharpened tungsten probe and an optical microscope. We have used transmission electron microscopy (TEM) to determine the structural characteristics of the Bi_2_Te_3_ NRs. Low-magnification TEM reveals a typical width of ~100–230 nm and a length exceeding 10 μm (Fig. 1a). High-resolution TEM confirms the single-crystallinity, with an atomic spacing of 2.2 Å (Fig. 1b, marked with arrows) along the 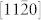 direction, similar to previously grown single-crystal Bi_2_Te_3_ NRs^21^. The Fourier transform (FFT) of the high-resolution TEM image reveals a high-quality single-crystalline structure with hexagonal symmetry (inset of Fig. 1b). We have also performed Raman spectroscopy with a 532 nm excitation laser on Bi_2_Te_3_ NRs (placed on SiO_2_/Si substrates). Figure 1c shows a representative spectrum. We observe three Raman peaks at 62 cm^−1^, 104 cm^−1^ and 137 cm^−1^, in excellent agreement with the Raman optical phonon modes (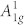, 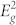 and 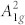) previously measured in bulk crystals^27^ and exfoliated thin flakes^28^,^29^ of Bi_2_Te_3_, further confirming the good crystalline quality of our NRs. Fig. 1d shows the atomic force microscope (AFM) image of a typical NR with thickness ~ 30 nm and width ~130 nm. Most of the electronic transport data presented below (unless otherwise noted) are measured from this representative Bi_2_Te_3_ NR, which is transferred onto a 500-μm-thick STO substrate (with very high relative dielectric permittivity κ at low temperatures^30^) and fabricated into a back gated field effect device (device #1). Similar results have also been obtained in several other devices measured.

## Temperature dependence and field effect

Effective gate control of topological surface states is highly desired for investigations of novel quantum transport and many device applications of TIs^4^,^21^. The optical image of device #1 and a schematic of its cross section are shown in the lower and upper insets of Fig. 2a, respectively. Figure 2a also shows the ambipolar field effect measured in the NR at 2K, where the decreasing backgate voltage (V_g_) tunes the carriers from n-type to p-type, with the resistance (R) peaking at the charge neutrality point (V_CNP_ ~ −17 V). We note that V_CNP_ should be above the Dirac point, which is shown to be buried inside the BVB from previous ARPES measurements on bulk Bi_2_Te_3_. The typical gated R modulation ratio is ~ 3–10 in our field effect devices. For V_g_ = 0 V, the temperature (T) dependence of the resistance (R(T)) of the Bi_2_Te_3_ NR device (Fig. 2b) shows a metallic behavior (R decreases with decreasing T), as previously observed in highly doped samples with bulk-dominated conduction^9^. However, for V_g_ = −6.5 V and −15 V, we observe an insulating behavior (R increases with decreasing T, due to bulk carrier freeze-out) for 10 K < T < 30 K with R saturating at a V_g_-dependent value (R^sat^) for T < 10 K. This R^sat^ corresponds to E_F_ inside the bulk bandgap and the low-T metallic conduction of topological surface states (TSS, see band schematic in the inset of Fig. 2b). For V_g_ = −22 V and −30 V (V_g_ < V_CNP_), in addition to the insulating behavior and low-T R^sat^, we observe R(T) peaks at T ~ 10 K. This peak resistance is similar to the peak resistance measured at V_g_ = V_CNP_ from the field effect (Fig. 2a, see also Fig. S1a, note the peak value can vary slightly after thermal cyclings), and is attributed to the strong enhancement of κ of STO^30^ at low T, such that E_F_ is lowered to cross CNP and further into the bulk valence band (VB) as T decreases (see also Fig. S1b, and its inset demonstrating *T*-dependence of V_CNP_). Our data shows that at low T, we can use V_g_ to tune E_F _(schematically represented with dashed lines in the inset of Fig. 2b) all the way from the conduction band (CB) to the TSS then to the VB. The measured R(T) suggests that bulk carriers in our Bi_2_Te_3_ NRs can be suppressed with V_g_ to realize a bulk insulating regime where surface can contribute dominantly to the conduction, and this is further corroborated with magnetotransport measurements presented later. A bulk metallic-to-insulator transition has also been observed in our Bi_2_Te_3_ NRs fabricated on SiO_2_/Si (eg., Fig. S2, device #4), while we have focused more on devices with STO backgate as they are found to generally give stronger gate tuning for ambipolar field effect^31^.

## Aharonov-Bohm Oscillations

When an external magnetic field (B) is applied along the length of the NR, the low-T magnetoconductance (G(B)) displays periodic oscillations (Fig. 3a) in B, commonly known as Aharonov-Bohm (A-B) oscillations (or “h/e oscillations”) with a characteristic period (ΔB = Φ_o_/A), where Φ_o_ = h/e is the flux quantum, A is the NR cross-sectional area (width×height), h is Planck's constant and e is the electron charge. In contrast, for bulk carriers there are impurity-dependent loops with no well-defined cross sectional area, resulting in universal conductance fluctuations (UCF), with non-periodic B-field dependence. A-B oscillations were previously observed in Bi_2_Te_3_ and Bi_2_Se_3_ NRs^21^,^32^. In order to probe the surface states, we measure G vs. parallel B-field from device #1 (Fig. 3a) at four representative V_g_'s. For V_g_ = −10, −11 and −13 V, periodic oscillations are clearly observed in Fig. 3a with ΔB ~ 1T (vertical dashed lines). This ΔB agrees well with Φ_o_/A = 1.06 T for our device, therefore these oscillations are the A-B oscillations, with G maxima at integer multiples of Φ_o_ (top axes). However for V_g_ = 4 V (E_F_ inside CB), the oscillations become non-periodical and they are attributed to UCFs due to bulk carriers.

**Figure 1:**
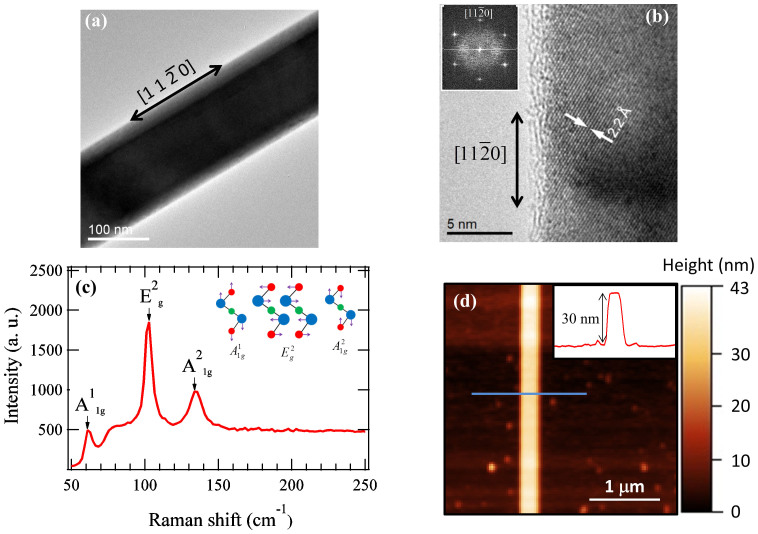
Material characterizations of Bi_2_Te_3_ nanoribbons. (**a**) Transmission electron microscope (TEM) image of a 150 nm wide Bi_2_Te_3_ nanoribbon (NR). NRs grow along the 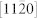 direction. (**b**) High-resolution TEM image, with the corresponding Fourier transform depicted in the inset. The obtained lattice spacing of 2.2 Å is consistent with the lattice spacing of 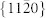 planes^16^,^26^. (**c**) A representative Raman spectrum (measured with a 532 nm laser) showing characteristic Raman peaks (labeled) similar to those observed in bulk Bi_2_Te_3_ (inset depicts corresponding phonon modes^27^,^28^) (**d**) Atomic force microscope (AFM) image of a 130 nm wide Bi_2_Te_3_ NR (device #1 before fabrication) on a SrTiO_3_ (STO) substrate. A thickness of 30 nm is extracted from the AFM line profile (inset, measured along the blue line in the AFM image).

The FFT of G (with a polynomial background subtracted) vs. 1/B for V_g_ = −10 V and T = 0.3 K is depicted in the left inset of Fig. 3a. We observe a dominant peak (~0.98 T^−1^), which is the period of the A-B oscillations. This period corresponds to an area A ~ 4000 nm^2^, again in good agreement with the AFM-measured cross-sectional area of the NR (~3900 nm^2^) of device#1 (Fig. 1d). The right inset of Fig. 3a displays the T-dependence of the amplitude (from FFT) of the A-B oscillations for V_g_ = -10 V, where a *T*^−1/2^ dependence is observed (other V_g_'s show similar behavior). Such a T^−1/2^ dependence is consistent with previous work in TI NRs^21^,^32^ and has also been observed in diffusive metallic^33^ and semiconducting rings^34^. We have also observed AB oscillations in two other devices (#2-3) with different cross sectional areas (A = 19800 and 3600 nm^2^ respectively). The measured period ΔB vs. V_g_ are depicted in Fig. 3b, along with the data from device#1. We observe that ΔB is independent of V_g_, but instead is controlled by A such that the product ΔB•A (magnet flux corresponding to ΔB) is ~ h/e (flux quantum) for all 3 devices (inset of Fig. 3b), confirming these oscillations are A-B oscillations of surface carriers.

## Shubnikov-de Haas oscillations

Figure 4a shows the magneto resistance (R(B)) vs. B-field (perpendicular to the NR surface and the substrate), measured for device #1 at T = 1.5 K and V_g_ = −8 V, where small oscillations are observed. The top inset of Fig. 4a displays ΔR (R(B) with a smooth polynomial background subtracted) at high B-field vs. 1/B, where periodical oscillations in 1/B are clearly observed. These oscillations are Shubnikov-de Haas (SdH) oscillations due to the formation of Landau levels (LL) in high magnetic fields. We take the FFT of ΔR (bottom inset of Fig. 4a) to find the frequency of oscillations (B_F_) ~ 55.4 T. The Fermi momentum (*k*_F_) and carrier density (*n*_SdH_) can be obtained from 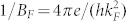 and 
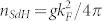
 respectively, where g = 1 is the degeneracy for spin polarized surface state (we attribute the SdH as coming from one surface, most likely the back-gated bottom surface, whereas another surface has much lower mobility and less conduction^9^,^10^,^35^). For the data shown in Fig. 4a, we obtain *n_SdH_* = 1.27 × 10^12^ cm^−2^ and k_F_ = 0.028 Å^−1^. We extract the phase of the SdH oscillations (which will correspond to a Berry's phase) following the standard procedure by plotting the integer Landau level index (n) vs. 1/B (Landau level fan diagram), as shown in Fig. 4b for several V_g_'s. An integer n is defined at ΔR minima (open markers) and n + 0.5 is defined at ΔR maxima (solid markers). The assignment of integer n's to ΔR *minima*, similar to the convention used in previous SdH studies in TINRs, is appropriate given the relatively large surface contribution to the conductance in our ultrathin NR samples (see a good discussion in ref. 36, also see Fig. S5). It is clearly seen that n fits (shown as lines) linearly with 1/B (n = *B_F_*/*B* + *β*), the slope (B_F_) varies with V_g_ and we extrapolate β ~ 0.5 for all V_g_'s (right inset of Fig. 4b, noting SdH oscillations were only observable in the range of V_g_ from −8 to −13 V with n-type TSS carriers). This β ~ 0.5 is further confirmed by a direct fitting (an example shown as the black solid line in Fig. 4a top inset) of the SdH oscillations to the low-T theoretical expression 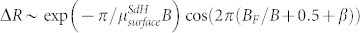 (yielding also B_F_ and SdH mobility 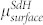 consistent with values from FFT/LL analysis above and SdH analysis discussed below). We have also plotted B_F_ (∝ *n_SdH_*) vs. V_g_ in the left inset of Fig. 4b, where the expected linear dependence from the effect of gating is observed. The LL indices for both Schrodinger and Dirac fermions can be linearly fitted to 1/B. However, it is well known that β = 0 for Schrodinger fermions and β = 1/2 for massless Dirac fermions, with the Berry's phase given by 2π β (Refs. 13,37,38,39). Such a prominent 1/2-shifted SdH effect provides a strong transport evidence of the spin-helical topological surface state Dirac fermions with the nontrivial Berry's phase π.

The amplitude of the SdH oscillations decreases with increasing T (while at each V_g_, we found that *n*_SdH_ does not change substantially with T in the range of temperature where SdH is observed). By fitting the T-dependence of the SdH oscillation amplitudes to the Lifshitz-Kosevich formula (Methods, and an example in Fig. 4c inset) we extract the cyclotron frequency (*ω_C_* = *eB*/m*) at the B-field position of a given R_xx_ minimum, corresponding to the N^th^ LL. At each V_g_ (with corresponding *n_SdH_*), the effective mass (m*) is obtained from the linear fit of ω_C_ vs. B-field. The extracted m* vs. *n_SdH_* (Fig. 4c) is not constant, rather proportional to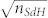, a distinct character for massless Dirac fermions previously demonstrated in graphene^38^,^39^. The calculated Fermi velocity (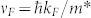) is nearly independent of *n_SdH_* (inset of Fig. 4d), with a mean value (〈*v_F_*〉 ~ 5 × 10^5^ *m*/*s*), in reasonable agreement with *v_F_* measured by ARPES in Bi_2_Te_3_ bulk crystals^6^ (*v_F_* = 4.1 × 10^5^ *m*/*s*). Figure 4d shows the corresponding Fermi energy (*E_F_* = m*〈*v_F_*〉^2^) as well as the m* vs. *k*_F_, where a linear dependence of *E_F_* (and m*) with *k*_F_ is demonstrated (dashed line represents a calculated linear dependence 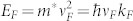 using *v_F_* = 〈*v_F_*〉 as constant Fermi velocity).

The density-independent *v_F_*, gate-tunable 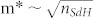 and therefore the linear band dispersion (E_F_ vs. *k*_F_) obtained in our experiments are in good agreement with the linear energy dispersion measured by ARPES in bulk Bi_2_Te_3_^6^,^7^, and are clear evidences that carriers in Bi_2_Te_3_ NRs behave as massless Dirac fermions in electronic transport.

**Figure 2:**
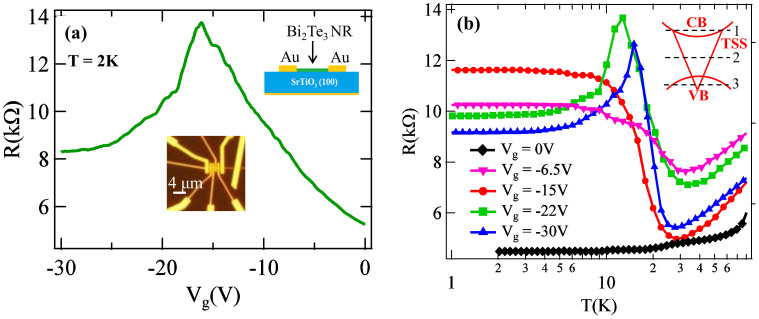
Gate-tuned bulk metal-insulator transition and ambipolar field effect. (**a**) Ambipolar field effect (4-terminals resistance R vs. V_g_), measured at T = 2 K. The upper (lower) inset depicts a schematic cross section (optical image, top view, with the inner electrodes used to measure the voltage separated by 850 nm) of the fabricated Bi_2_Te_3_ NR device #1 on a 500 μm-thick STO substrate. (**b**) Temperature (plotted in log scale) dependence of R of device #1 measured at five gate voltages (V_g_). Inset: schematic of the band diagram of Bi_2_Te_3_. The horizontal dashed lines depict 3 representative locations of the Fermi energy (E_F_), intercepting with the bulk conduction band (CB), topological surface states (TSS) and bulk valence band (VB), respectively. Note the Dirac point (DP) is buried inside the VB, thus only n-type TSS carriers are accessible inside the bulk bandgap.

**Figure 3:**
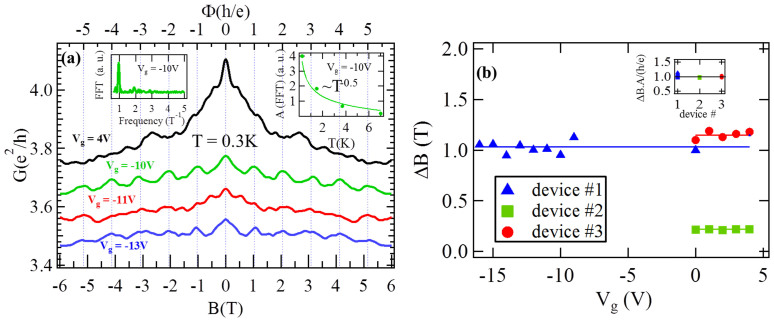
Aharonov-Bohm oscillations of the surface carriers. (**a**) Magnetoconductance (G) vs. magnetic field (B, with the corresponding magnetic flux Φ plotted on the top axis) applied parallel to the NR length (device #1) at 4 different V_g_'s (curves vertically offset for clarity, except for V_g_ = -13 V) measured at T = 0.3 K. Left inset: FFT of G (V_g_ = -10 V) vs. 1/B, after a background subtraction in G. Right inset: temperature dependence of the FFT amplitude for the h/e oscillations at V_g_ = -10 V; a fitting with a T^-0.5^ dependence is plotted as a solid line. (**b**) Period (ΔB) of oscillations vs. V_g_ for three devices with different cross section areas (A = 3900, 19800 and 3600 nm^2^ for devices 1, 2 and 3 respectively). The inset depicts magnetic flux (ΔB•A, in units of h/e) for these 3 devices (including all data points measured at different V_g_'s shown in the main panel).

We have extracted the (high-magnetic-field) quantum lifetime (τ_q_) of surface carriers for each V_g_ (Fig. S3 shows 2 examples) from the slope of 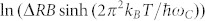 vs. 1/B (so called “Dingle analysis”, see Methods and Supplemental Materials). We also calculated the corresponding high-field quantum mean free path (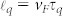), SdH mobility 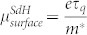, and SdH conductivity 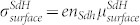 (See Fig. S4) of the surface carriers that give SdH oscillations at T = 1.5 K. Since it is expected that τ_q_<τ_tr_, where τ_tr_ is the transport lifetime (contributed mainly by large-angle scattering rather than by all scatterings as in τ_q_)^40^ of surface state carriers at B = 0 T, the calculated 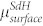 and 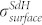
provide *lower bounds* for the (zero-field) transport mobility (*μ_surface_*) and conductivity (*σ_surface_*) of the corresponding surface. We have plotted the (dimensionless) ratio 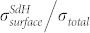 vs. the gate voltage in Fig. S5, where *σ_total_* = (*L*/*W*)/*R* is the total (2D sheet) conductivity of the NR (measured at zero-magnetic-field, with L, W and R being the length, width and 4-terminals resistance of the TI NR, respectively; noting all conductivities in this paper are 2D sheet conductivities). For the reasons discussed above (due to τ_q_<τ_tr_) and the fact that the contribution from only one surface (that gives SdH oscillations) is included in 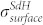, this ratio (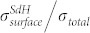) *underestimates* the actual surface contribution to the total conductance. The large value of 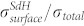 (reaching ~0.5 at V_g_ ~ -8 V) is notable, confirming that the surface can make substantial or even dominant contribution (manifested in the residual saturating metallic resistance seen in the R vs T in Fig. 2b) to the total conductance at low T (where bulk is insulating at this V_g_). The reduction of 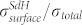 for decreasing V_g_ (towards V_CNP_) might be related to the shape of the bandstructure of Bi_2_Te_3_ near the Dirac point (DP), which is buried inside the bulk VB (Fig. 2b inset).

## Weak antilocalization and localization

Weak antilocalization (WAL) appears as a negative magnetoconductivity (Δσ(B) = σ(B) – σ(B = 0 T)) with a sharp cusp centered at zero B-field. It is commonly observed in TIs due to the spin-momentum locking resulting from strong spin-orbit coupling. The π-Berry's phase carried by the topological surface states of TIs leads to destructive interference between time-reversed paths, enhancing *σ*(*B* = 0*T*). The WAL cusp can be destroyed by applying a B-field that breaks the time reversal symmetry and π-Berry's phase^41^. We use the simplified Hikami-Larkin-Nagaoka formula^41^,^42^ to fit Δ*σ*(*B*): 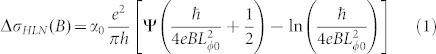
where Ψ is the digamma function, L_φ0_ is the phase coherence length, and α_0_ is a prefactor. Each coherent channel that carries a π-Berry's phase should give α_0_ = -1/2. In contrast, if the Berry's phase is absent, the destructive interference mentioned above would become constructive, resulting in weak localization (WL). The WL appears as a positive magnetoconductivity that can be similarly fitted to Eq. (1) with a positive α_0_. Figure 5a shows the magnetoresistance R(B) measured at 1.5 K at five different V_g_'s. By increasing V_g_ from −18 V to +14 V, we observe that R(B) transitions from exhibiting a dip (indicating negative Δσ(B), and WAL) to a peak (indicating positive Δσ(B), and WL) near zero B field. Figure 5b displays the anti-symmetrized Δσ(B) (extracted from the R(B)) vs. B-field at these V_g_'s, where the gate induced transition from negative to positive Δσ(B) (WAL to WL) is clearly observed. Also, we note that the amplitude of the WAL or WL cusp depends on V_g_. We first focus on the gate-tunable WAL for V_g_ < 0 V. Using equation 1, we fit Δσ (B) (for B < 2 T) at different V_g_'s and extract the gate-dependent α_0_ and L_φ0_ (Fig. 5c). We observe α_0_ peaks ~ −1 for V_g_ ~ −18 V (~V_CNP_) and approaches −0.5 for either V_g_ > −12 V (E_F_ approaching the CB) or V_g_ ~ −30 V (E_F_ near the VB). The parameter α_0_ is related to the number of coherent conduction channels *A* = 2|*α*_0_|. The transition from α = −0.5 (A = 1, away from CNP) to α = −1 (A = 2, near CNP) tuned by V_g_ reflects the transition of the system from a single coherent channel (with bulk and TSS strongly coupled together) to two decoupled coherent channels surrounding the two surfaces (as bulk conduction is suppressed by the gate^22^,^25^,^42^,^43^). Also, L_φ0_ displays a minimum of ~ 62 nm for V_g_ ~ V_CNP_. For V_g_ ≥ 2 V, Δσ(B) transitions into positive and weak localization (WL) behavior, although a small WAL (negative Δσ(B)) can still be observable at very low B fields (B < 0.2 T, as shown in Fig. 5b), reflecting a competition (mixed contributions) between WAL and WL. We perform a two-component fit for Δσ(B) using equation 2, derived by Lu-Shi-Shen (LSS)^44^, to extract the WAL (α_0_, L_φ0_) and WL (α_1_, L_φ1_) contributions, 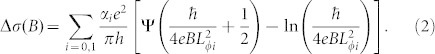
In order to obtain unique fitting results for the WAL and WL parameters (using equation 2), we fix the WAL prefactor α_0_ = −0.5 (a reasonable choice given Fig. 5c shows α_0_ saturates at ~−0.5 for V_g_ >−12 V). We observe (Fig. 5d) that as V_g_ increases from 2 V to 14 V, both the WL prefactor α_1_ (~1) and WAL phase coherence length L_Φ0_ (~ 120 nm) remain relatively constant, while the WL phase coherence length L_Φ1_ moderately increases from 46 to 76 nm. Transitions from WAL to WL in TIs have previously been observed in magnetically doped TIs^45^ and extremely thin TI flakes (4–5 nm thick)^46^,^47^, and are attributed in both cases to gap opening in the surface states (i.e., DP destroyed)^44^,^48^. It has been theoretically predicted that TI can also display a competition between WAL and WL, *without* involving surface gap opening and destroying the DP, if E_F_ is close to the transition region between TSS and the bulk bands (CB or VB)^24^,^25^. It has been pointed out that as E_F_ moves from the TSS to near the bottom of CB, the Berry's phase decreases from of π to 0 (due to change in the pseudospin texture, where the bulk bandgap can act as a fictitious Zeeman field perpendicular to the spin-orbit field), giving rise to a WAL to WL transition^24^,^25^. Observing such WAL to WL transition as predicted^24^,^25^ is another piece of evidence that we are actually observing topological surface states transport.

## Conclusions

In conclusion, we have achieved ambipolar field effect on single crystal Bi_2_Te_3_ NRs using STO as substrate and backgate dielectric. We are able to use the gate to tune the electronic transport from being dominated by the metallic bulk to a bulk-insulating regime such that surface state contributes mostly to the conduction at low temperatures. We observed gate-controlled A-B oscillations of surface carriers. We have also measured gate-controlled SdH oscillations with an extracted π-Berry's phase, m* proportional to square root of carrier density, and nearly constant *v_F_*, providing direct evidence of the Dirac fermion nature (linear E_F_ vs. k_F_) of the topological surface states. Also, a gate-tunable WAL is observed and the extracted number of coherent conduction channels is found to peak around 2 (corresponding to 2 decoupled channels surrounding 2 surfaces) for V_g_ ~ V_CNP_. Finally, we observe a competition between WAL and WL for V_g_ > 0 V, consistent with a predicted collapse of Berry's phase and change of pseudospin texture as E_F_ approaches the CB. The gate-tunable topological surface states found in our samples pave the way towards topologically protected nanoelectronic and spintronic devices and possible applications in topological quantum computing with Majorana fermions. During the revision of the previous version of our manuscript, we became aware of another experiment on Bi_2_Te_3_ that also reported the observation of linear dispersion and relativistic mass in SdH oscillations^49^.

## Methods

### Nanoribbons synthesis and transfer

Bulk Bi_2_Te_3_ (99.999%, Alfa Aesar) was placed in the center of a single-zone furnace (TF55035A-1, Lindburg® BlueM®) and SiO_2_ covered Si substrates were placed downstream. The temperature was slowly ramped (1–2°C/min) to ~480°C under flowing Ar (~75 cm^3^/min, 99.999% purity) and pressure was maintained in the range of 20–70 Torr for the duration of the synthesis. Growth times were on the order of 1–3 hours.

### Transmission electron microscopy (TEM)

Transmission electron microscopy (200 kV, JEM-2010F, JEOL Ltd.) analysis of several NRs showed growth along the 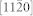 direction and energy dispersive X-ray spectroscopy indicated excess tellurium (66 ± 2 at.% Te). The tips of the NRs were observed to be catalyst free with 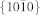 facets.

### Raman Spectroscopy

Raman spectroscopy was performed using a Horiba Jobin Yvon Xplora confocal Raman microscope. The wavelength of the excitation laser was 532 nm and the power of the laser incident on the sample was kept below 200 μW to avoid sample burning. The laser spot size was ~ 0.6 μm with a 100X objective lens (numerical aperture = 0.90). The spectral resolution was 1.0 cm^−1^ (using a grating with 2400 grooves/mm) and each spectrum was an average of 3 acquisitions (5 seconds of accumulation time per acquisition).

### Atomic Force Microscopy (AFM)

The width and thickness of the samples was measured by a NT-MDT NTEGRA Prima multifunctional atomic force microscope in tapping mode configuration. In order to avoid any damage or NRs displacement, we use a small cantilever driving amplitude and set point amplitude corresponding to 75% of the freely resonant magnitude. The silicon probes used are ACTA series from AppNANO, with a nominal 40 N/m spring constant, cantilever length of 125 μm and 6 nm radius of curvature.

### Device fabrication and electronic transport measurements

Our STO substrates are single side polished (100) from Shinkosha Ltd. The electrical contacts (Cr/Au, 5 nm/70 nm, e-beam evaporated) to Bi_2_Te_3_ NRs were patterned by e-beam lithography. Right before the metal deposition, a short etch (20 sec.) in a dilute solution of sulfuric acid (H_2_SO_4_:H_2_O, 1:10) is performed to remove native oxide from the surface. After the metal evaporation, the device is mounted and wirebonded to a ceramic chip carrier. Cr/Au (10/70 nm) is deposited on the backside of STO (backgate electrode). All the electrical measurements are performed in a top loading Helium-3 system (Heliox TL system, Oxford Instruments). The resistances were measured by 4-terminals method and using low frequency lock-in detection (PAR124A), with a driving current of ~1 μA.

### Temperature dependence of the Shubnikov de Haas oscillations and effective mass calculation

**Figure 4:**
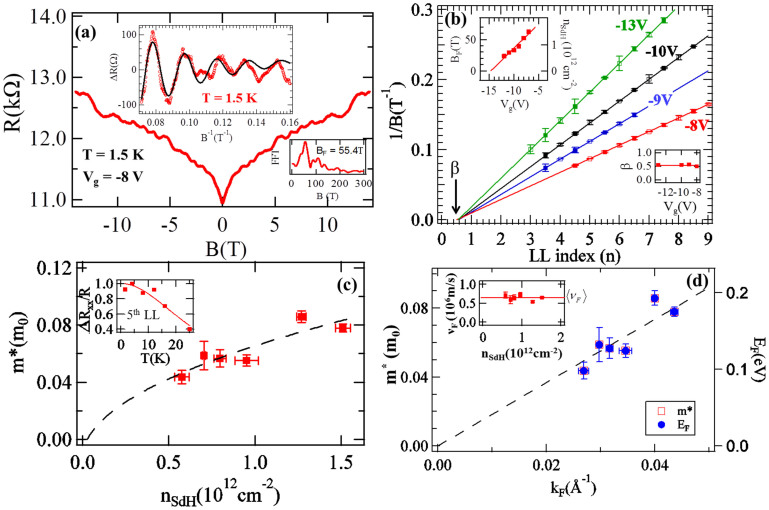
Shubnikov-de Haas (SdH) oscillations of surface state Dirac fermions showing linear E-k dispersion. (**a**) Representative magnetoresistance (R vs. B) with B applied perpendicular to the NR plane, measured at V_g_ = -8 V and T = 1.5 K for device #1. Top inset: ΔR (R with a background substracted) vs. 1/B (red, with a fit shown as black curve and FFT shown as bottom inset). (**b**) 1/B vs. LL index (fan diagram) at different V_g_'s. Right inset: β (intercept of LL index for 1/B →0 as extracted from the fan diagram) vs. V_g_. Left inset: SdH oscillation frequency (B_F_, left axis, inverse slope of 1/B vs. LL index) and density n_SdH_ (right axis) vs. V_g_ (solid line is a linear fit) (**c**) Extracted effective mass (m*) vs. carrier density (n_SdH _ = eB_F_/h), where m* is extracted by equation (3) from the temperature dependence of the SdH oscillations. The dashed line is a fit to 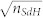 behavior. The inset shows ΔR/R vs. T, for LL index = 5. (**d**) Effective mass m* (left axis) as well as Fermi energy (*E_F_* = m*〈*v_F_*〉^2^) vs. Fermi momentum (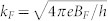), where 〈*v_F_*〉 is the average (and mostly constant) Fermi velocity (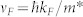) over the n_SdH_ range measured (inset). Black dashed line represents a calculated linear dependence using 〈*v_F_*〉 as Fermi velocity.

**Figure 5:**
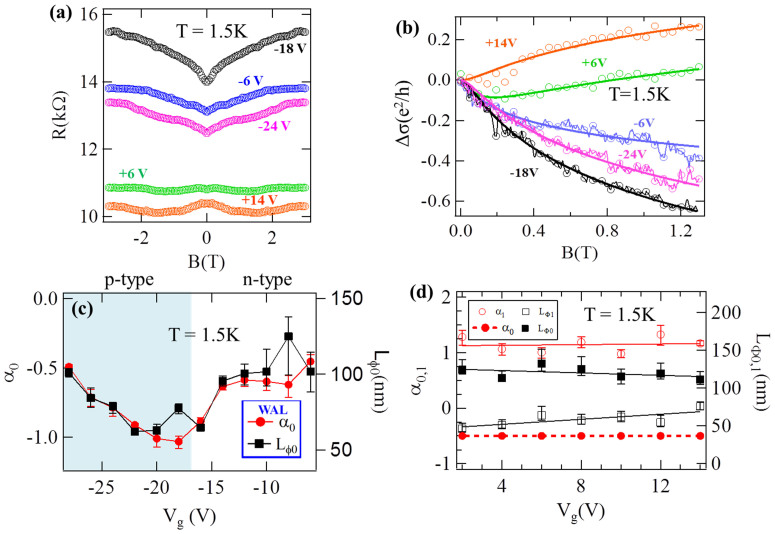
Gate-tuned weak antilocalization (WAL) and transition to weak localization (WL). (**a**) Magnetoresistance (R vs. B, with B applied perpendicular to the NR plane) at 5 different V_g_'s at T = 1.5 K for device #1. (**b**) Magnetoconductivity (Δσ(B), where Δσ(B) = σ(B) - σ(B = 0 T)), normalized by e^2^/h. Solid lines depict fitting to HLN equation (see text). (**c**) Weak antilocalization (WAL) prefactor α_0 _(left axis) and phase coherence length L_Φ0 _(right axis), both extracted using the HLN fit, as functions of V_g_ (for negative V_g_). (**d**) Weak localization (WL) prefactor α_1_ (left axis) and phase coherence lengths (right axis) for WAL and WL contributions (solid lines are guides to the eye), extracted from the two-component HLN fit, with fixed α_0_ = -0.5, for V_g_ > 2 V.

We fit the T-dependence of SdH oscillation amplitude (ΔR) to the Lifshitz-Kosevich theory^50^: 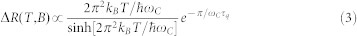
Where ω_C_ and τ_q_ are the fitting parameters and B is the magnetic field position of the N^th^ minimum in ΔR; k_B_ is the Boltzmann's constant; *ω_C_* = *eB*/m* is the cyclotron frequency (and 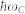 is the cyclotron energy gap for the N^th^ LL); m* is the effective mass of the carriers and τ_q_ is the quantum lifetime. The ω_C_ is calculated from the fitting of the relative amplitude ΔR/R(B = 0T) as a function of T using equation (3) (inset of Fig. 4c) for different LLs. The m* is extracted from the slope of ω_C_ vs. B for a given V_g_.

## Author Contributions

L.A.J. designed and fabricated the devices and carried out the measurements. M.T.P. and L.S. synthesized the Bi_2_Te_3_ nanoribbons and performed structural analysis. L.P.R. and Y.P.C. contributed to the measurements and analysis. Y.P.C. supervised the research. L.A.J. and Y.P.C. wrote the paper with contributions from all other co-authors.

## Supplementary Material

Supplementary Information Supplementary Info File #1
