# Supplementary material for: Gate Tunable Relativistic Mass and Berry's phase in Topological Insulator Nanoribbon Field Effect Devices
Source: Sci Rep. 2015 Feb 13;5:8452. doi: 10.1038/srep08452 (PMC4326695; doi:10.1038/srep08452)
Supplement: Supplementary Information — Supplementary Info File #1 [file srep08452-s1.docx]

**Supplementary Information**

**Gate Tunable Relativistic Mass and Berry’s phase in Topological Insulator Nanoribbon Field Effect Devices**

Luis A. Jauregui^1,2^, Michael T. Pettes^3,˫^, Leonid P. Rokhinson^4,1,2^, Li Shi^3,5^, Yong P. Chen^1,4,2,*^

^1^ Birck Nanotechnology Center, Purdue University, West Lafayette, IN 47907

^2^ School of Electrical and Computer Engineering, Purdue University, West Lafayette, IN 47907

^3^ Department of Mechanical Engineering, University of Texas at Austin, Austin, TX 78712

^4^ Department of Physics and Astronomy, Purdue University, West Lafayette, IN 47907

^5^ Materials Science and Engineering Program, University of Texas at Austin, Austin, TX 78712

^˫^ Current address: Department of Mechanical Engineering, University of Connecticut, Storrs, CT 06269

* To whom correspondence should be addressed: [yongchen@purdue.edu](mailto:yongchen@purdue.edu)

Figure S1a depicts the field effect of R at T=0.3K for device #1 in different cool downs. The measurements were in the following order: black, blue and green, measured after measuring R vs. T for V_g_ = 0, -30 and -22V respectively. The magenta dashed curve was a repeat measurement for the green curve to show the reproducibility of R vs. V_g_ in the same cool down. We observe that V_CNP_ and the peak resistance have moderate changes with different cool-downs (which may reflect re-arrangement of impurities inside TI and the STO substrate). Figure S1b depicts the field effect of R for device #1 (main text) at different T’s. The V_CNP_ changes from V_CNP_ ~ -15V (T = 4K) to V_CNP_ ~ -40V (T = 30K). The change of V_CNP_ with temperature can be attributed to the significant reduction of STO gate capacitance as temperature increases from 4K to 30K [^30^](#_ENREF_30).

Figure S2, shows the R(T) for another TI NR device (device #4) on SiO_2_ (300nm thick)/doped Si substrate (with gate capacitance largely T-independent) for 2 different V_g_’s. Data at V_g_ = 0V show a metallic behavior (R decreases with decreasing T). However, for V_g_ = -70V, we observe an insulating behavior (R increases with decreasing T) for T > 70 K with R saturating (R^sat^) for T < 70 K. This corroborates the gate-tuned metal to insulator transitions in the bulk of Bi_2_Te_3_ NRs in devices fabricated on STO substrate (Fig. 2b).

*Extraction of quantum lifetime, SdH mobility and SdH conductivity*

Figure S3a (S3b) displays vs. 1/B at V_g_ = -8V (V_g_ = -12V) at T = 1.5K. The τ_q_ is extracted from the slope of  vs. 1/B, where τ_q_ varies from 0.5 x10^-13^ sec (V_g_ = -12V) to 1.3x10^-13^ sec (V_g_ = -8V) in our experiments.  The surface mobility ($\mu_{surface}^{SdH}=\frac{e\tau_{q}}{m*}$) is found to be $\mu_{surface}^{SdH}$ ~ 2,000 – 3,000 cm^2^/Vs and quantum mean free path () ~ 30 - 50 nm, as depicted in Fig. S4. The gate-dependent ratio between high-B surface SdH conductivity ($\sigma_{surface}^{SdH}=en_{Sdh}\mu_{surface}^{SdH}$, which is smaller than the surface transport conductivity at zero-B) and total conductivity ($\sigma_{total}$, measured at zero-B) is plotted in Fig. S5, showing a significant value with notable enhancement near CNP, consistent with the expected large surface to bulk conductance ratio (which is bounded from below by the plotted ratio) near CNP.


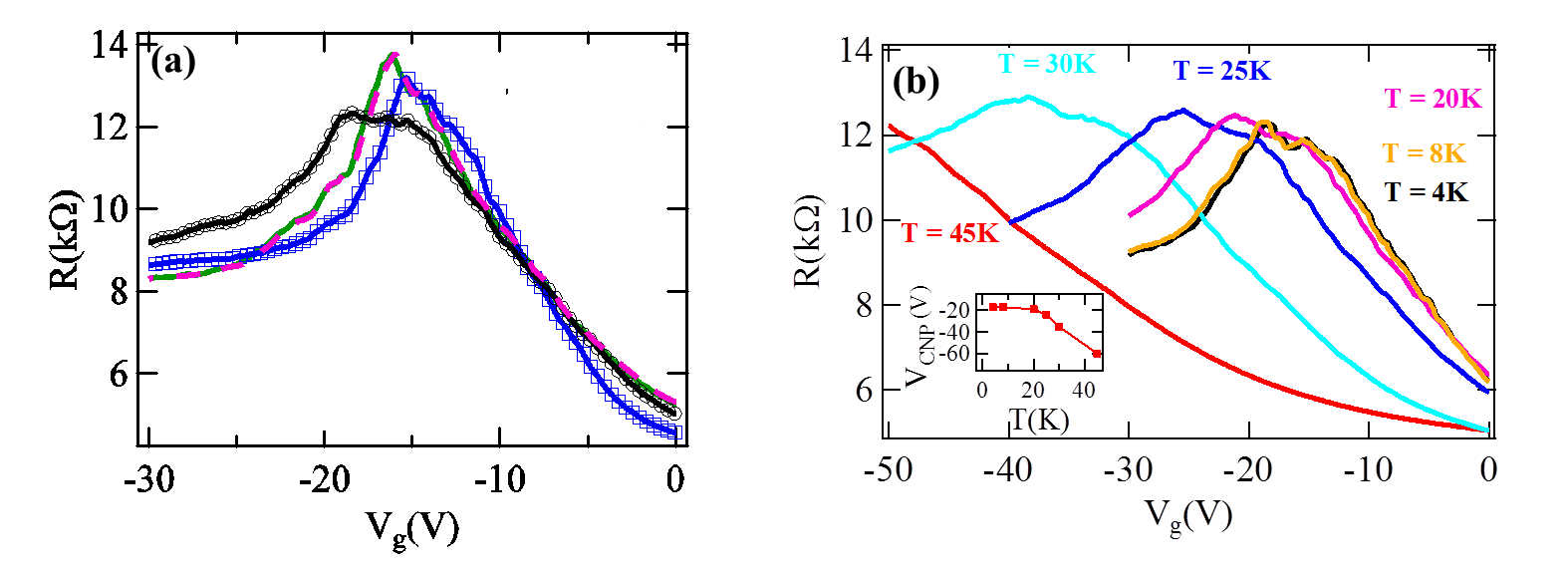


Figure S1. **Field effect and T-dependence of Bi_2_Te_3_ NR on STO (device #1). (a)** R vs. V_g_ measured for device #1 at T = 0.3 K, each curve was taken after the thermal cycling caused by the measurements of R vs. T depicted in the main Fig. 2b. The measurements were in the following order: black, blue and green (magenta), measured after measuring R vs. T for V_g_ = 0, -30 and -22V respectively. The magenta dashed curve was a repeat measurement of the green curve to show the reproducibility of R vs. V_g_ in the same cool down. **(b)** Field effect of Resistance (R) measured at different temperatures for device #1. Inset: temperature dependence of the V_CNP_.


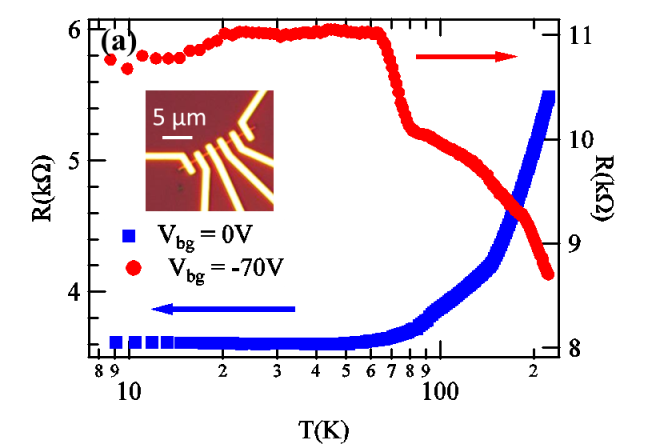


Figure S2. **Temperature dependence of the resistance of a Bi_2_Te_3_ NR on SiO_2_/Si. (a)** R vs. T measured from device #4 (Bi_2_Te_3_ NR of width = 330nm and thickness ~ 30nm on 300nm SiO_2_/doped Si) at two V_g_’s. The inset depicts the optical image of the device.


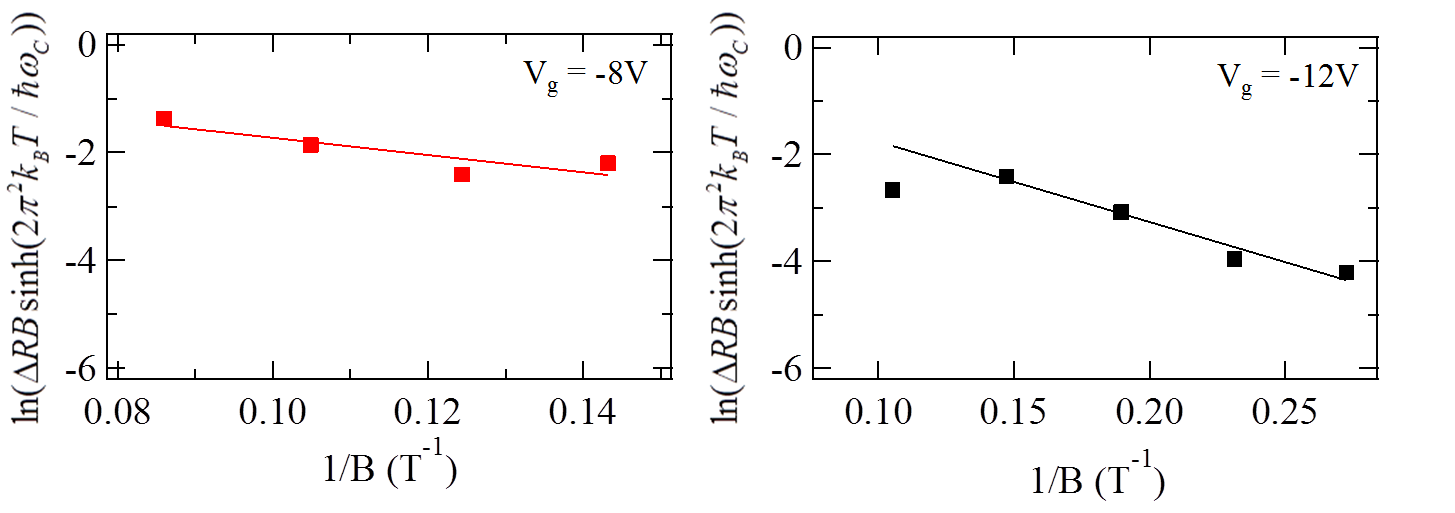


Figure S3. **Representative examples of the Dingle analysis used to obtain the quantum lifetime (τ_q_).** vs 1/B at V_g_ = -8V **(a)** and V_g_ = -12V **(b)** measured at T = 1.5K for device #1, where τ_q_ is obtained from the slope. The ω_C_ and ΔR are defined in Methods.


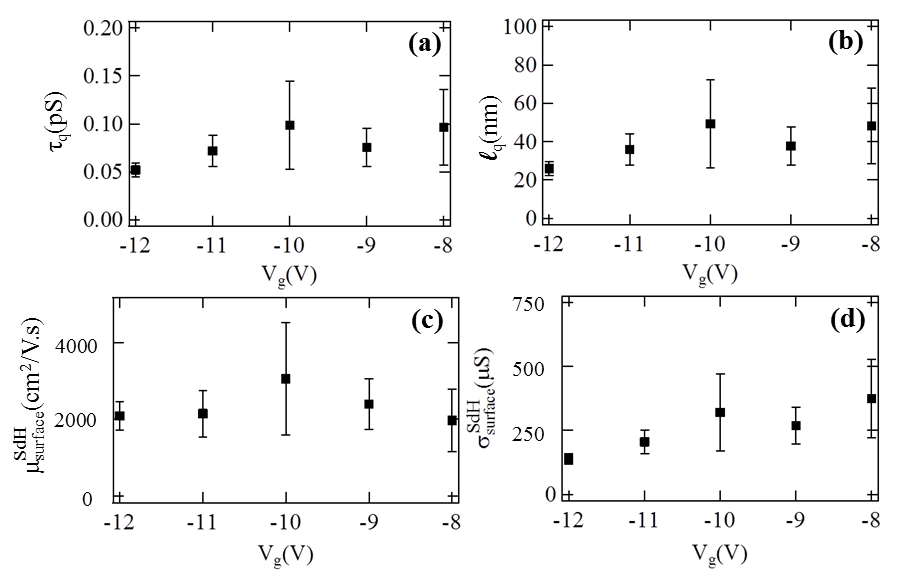


Figure S4. **Surface state quantum lifetime (a), quantum mean free path (b), high-field mobility (c) and high-field conductivity (d) extracted from the SdH oscillations,** extracted for 5 V_g_’s **(**for device #1 at T = 1.5K. Note the high-B (SdH) surface mobility ($\mu_{surface}^{SdH}$) and conductivity ($\sigma_{surface}^{SdH}$) are *lower bounds* for the surface mobility and conductivity measured at B = 0T respectively.


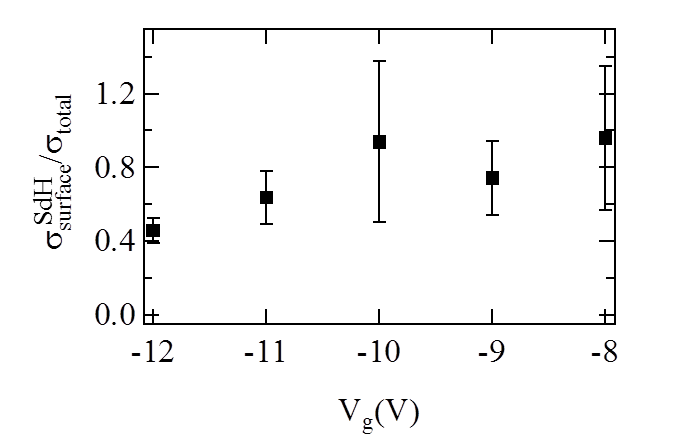


0.0

0.2

0.4

0.6

Figure S5. **Ratio of high-field surface conductance to zero-field total conductance.** Shown is $\sigma_{surface}^{SdH}/\sigma_{total}$ at T = 1.5K vs. V_g_ for device #1, where $\sigma_{surface}^{SdH}=en_{Sdh}\mu_{surface}^{SdH}$, is the quantum mean free path (= ν_F_τ_q_) , τ_q_ is the quantum lifetime of the surface states (Fig. S4), and $\sigma_{total}$ is the total conductivity of the NR at B = 0T. The plotted ratio is a lower bound for the surface-to-total conductance ratio at zero-B, as discussed in the main text.
